# Drought-tolerant *Desmodium* species effectively suppress parasitic striga weed and improve cereal grain yields in western Kenya

**DOI:** 10.1016/j.cropro.2017.03.018

**Published:** 2017-08

**Authors:** Charles A.O. Midega, Charles J. Wasonga, Antony M. Hooper, John A. Pickett, Zeyaur R. Khan

**Affiliations:** a International Centre of Insect Physiology and Ecology (*icipe*), P.O. Box 30772, Nairobi 00100, Kenya; b Biological Chemistry and Crop Protection Department, Rothamsted Research, Harpenden, Hertfordshire AL5 2JQ, UK

**Keywords:** Striga, Climate change, *Desmodium*, Drought-tolerance, Agricultural productivity

## Abstract

The parasitic weed *Striga hermonthica* Benth. (Orobanchaceae), commonly known as striga, is an increasingly important constraint to cereal production in sub-Saharan Africa (SSA), often resulting in total yield losses in maize (*Zea mays* L.) and substantial losses in sorghum (*Sorghum bicolor* (L.) Moench). This is further aggravated by soil degradation and drought conditions that are gradually becoming widespread in SSA. Forage legumes in the genus *Desmodium* (Fabaceae), mainly *D. uncinatum* and *D. intortum*, effectively control striga and improve crop productivity in SSA. However, negative effects of climate change such as drought stress is affecting the functioning of these systems. There is thus a need to identify and characterize new plants possessing the required ecological chemistry to protect crops against the biotic stress of striga under such environmental conditions. 17 accessions comprising 10 species of *Desmodium* were screened for their drought stress tolerance and ability to suppress striga. *Desmodium incanum* and *D. ramosissimum* were selected as the most promising species as they retained their leaves and maintained leaf function for longer periods during their exposure to drought stress conditions. They also had desirable phenotypes with more above ground biomass. The two species suppressed striga infestation, both under controlled and field conditions, and resulted in significant grain yield increases, demonstrating the incremental capability of *Desmodium* species in striga suppression. These results demonstrate beneficial effects of *Desmodium* species in enhancing cereal productivity in dry areas.

## Highlights

• Parasitic weeds and soil degradation constraint cereal production in Africa.
• Climate change aggravates these, reducing functionality of control approaches.
• Drought tolerant *Desmodium* spp. effectively control striga and improve cereal grain yields.
• Intercropping with these *Desmodium* spp. provide opportunity to improve cereal production in drier areas.

## 1 Introduction

Production of cereal crops, principally of maize (*Zea mays* L.) and sorghum (*Sorghum bicolor* (L.) Moench) in many regions of Africa is constrained by a complex of biotic and abiotic factors, with parasitic weeds of the genus *Striga* being among the most serious. There are about 23 species of *Striga* in Africa, of which *Striga hermonthica* (Del.) Benth., commonly known as striga, is the most socio-economically important constraint in cereal cultivation in eastern Africa (Gressel et al., 2004, Gethi et al., 2005). Infestation by striga causes grain yield losses of up to 100%, amounting to estimated annual losses of $40.8 million (Kanampiu et al., 2002), and these effects are most severe in degraded environments with low soil fertility and low rainfall, and in subsistence farming systems with few options for use of external inputs (Gurney et al., 2006).

Striga germinates close to its hosts in response to specific chemical signals from the root exudates of the host or certain non-host plants (Hooper et al., 2009), indicating an ingenious adaptation and integration with the hosts (Bebawi and Metwali, 1991). Following germination, the radicle grows and, when approaching the host root cells, undergoes haustoriogenesis giving rise to the functional attachment organ through which parasitism is initiated (Hooper et al., 2009, Hooper et al., 2015). The significant reductions in crop yields realized from striga infestations result from a series of physiological changes in the host plants following striga parasitism. These include weakening of the host, wounding of its outer root tissues and absorption of its supply of moisture, photosynthates and minerals (Tenebe and Kamara, 2002). There is also a “phytotoxic” effect expressed within days of attachment to its host whose underlying mechanism has not yet been established (Gurney et al., 2006).

Control of striga has been complicated by the abundant seed production by striga plant, longevity of the seed bank, and a complicated mode of parasitism. Nonetheless, a number of control options, such as imidazolinone resistant maize varieties whose seed is coated with imidazolinone herbicides to control striga, have been developed (Kanampiu et al., 2003). One of the most effective ways of managing striga is the use of forage legumes in the genus *Desmodium* (Fabaceae) as an intercrop which provides the key chemical components for inhibiting development of striga in the field (Khan et al., 2008, Midega et al., 2014). *Desmodium* spp. suppress striga through a combination of mechanisms, including abortive germination of striga seeds that fail to develop and attach onto the hosts’ roots (Khan et al., 2002, Tsanuo et al., 2003).

Climate variability has in the recent past become a serious threat to food production in SSA, largely due to the rainfed nature of much of the farming systems. Notably, there have been progressive increases in atmospheric temperatures, extended drought periods and reduced and erratic rainfall (Midega et al., 2015). Associated with these has been significant degradation and loss of arable land (Oldeman et al., 1990), cumulatively resulting in significant negative effects on food production, crop season length, and higher-order social impacts, including food insecurity (Sivakumar, 1993). There is thus a need to adapt effective cropping strategies such as the *Desmodium*-based system to climatic variabilities to ensure sustainable food production and environmental conservation (Midega et al., 2015). Our previous studies demonstrated effective control of the parasitic striga by the drought-tolerant *Desmodium intortum* (Mill.) Urb. Here, we report efforts to identify additional drought-tolerant *Desmodium* spp. to expand options available to smallholder farmers in agro-ecologies with varying degrees of drought and degraded soil conditions. Specifically, we sought to (i) establish drought tolerance in selected *Desmodium* spp., and (ii) evaluate their effectiveness in striga suppression, both under controlled and farmers’ fields in western Kenya.

## 2 Materials and methods

### 2.1 Plants

Seeds of accessions of various *Desmodium* species were obtained from the International Livestock Research Institute (ILRI) forage plants gene bank (Ethiopia), Desert Legumes Program - University of Arizona (USA), and the USDA-ARS Plant Genetic Resources Conservation Unit (USA). The accessions were initially planted at *icipe*-Thomas Odhiambo campus (*icipe*-TOC), Mbita Point (0°25′S, 34°12′E; 1200 m above sea level), western Kenya, and examined for above-ground allometries to allow for a pre-selection of candidate accessions with desirable phenotypes: high biomass and low growth habit (for use as intercrops). From the initial observation trials, 17 accessions representing 10 *Desmodium* species with desirable phenotypes were selected for screening for drought tolerance. These were *D*. *dichotomum* (Willd.) D.C., *D. tortuosum* (Sw.) D.C., *D. uncinatum* (used as check), *D. ramosissimum* G. Don., *D. repandum* (Vahl.) D.C., *D. distortum* (Aubl.) J.F. Macbr., *D. incanum* D.C., *D. grahamii* A. Gray, *D. heterophyllum* (Willd.) D.C. and *D. psilocarpum* Gray.

### 2.2 Screening *Desmodium* species for drought tolerance

#### 2.2.1 Growth environment and drought stress treatments

Single plants of the candidate accessions were grown in semi-restricted soil columns comprising white plastic bags measuring 60 cm deep and 26 cm wide filled with black cotton soils as the growth medium in a screen house at *icipe*-TOC. The soil columns were fitted with two 1-cm drainage holes at the base and mounted on 30 cm high wire mesh covered metallic benches to avoid direct contact with the ground. The minimum and maximum daily temperatures in the screenhouse during the period of the experiment averaged 17 °C and 35 °C, respectively. The plants were initially established and maintained under adequate soil moisture conditions for eight weeks and then subjected to 10 continuous weeks of exposure to three soil water regimes: severe drought stress (W3), moderate drought stress (W2) and a well-watered control (W1). In the severe water stress treatment, the plants were not watered for the 10 weeks. For the moderate drought stress treatment the plants received 125-mm of water uniformly distributed over the 10 weeks and delivered every two days through irrigation by hand. For the control treatment the plants were watered every two days to near field capacity for the duration of the trial.

Progression to wilting of above ground biomass of different *Desmodium* accessions exposed to 10 weeks of severe drought stress was visually monitored weekly and scored as either wilted or not. A plant was scored as wilted when signs of wilting were observed on leaves from the base of the plant to terminal whorl and shoots. At the end of the 10 weeks of trial the number of days an accession took to wilt was recorded.

#### 2.2.2 Leaf water content

Plant leaf water status as affected by the drought treatments was evaluated using methodologies adapted from Abraham et al. (2004) and Bouchabke et al. (2008) using a smaller number of accessions that had shown ability to withstand extended periods of drought, and with useful growth habits as above. These were *D. repandum*, *D. incanum* and *D. ramosissimum*, with *D. uncinatum* being included as a control check. Recently fully expanded *Desmodium* leaves were sampled weekly at 12:00pm, noon when evapo-transpiration peaked, starting at the end of the sixth week after the drought stress treatments commenced. Four leaf samples were sampled from each of the three water treatments for each of the *Desmodium* species and their fresh weights were individually recorded. The leaves were then put in separate paper envelopes and oven-dried at 85 °C for 24 h, after which the final weights of the dried leaves were measured. Leaf water content (LWC) was determined using the equation:

LWC=((FW−DW)/FW)×100

Where FW is leaf fresh weight and DW is dry weight of leaves after being oven-dried.

### 2.3 Leaf electrolyte conductance and cell membrane stability

Leaf electrolyte conductance was measured, using procedure adapted from Hirashima et al. (2009), to estimate ability of the four *Desmodium* spp. to accumulate leaf electrolytes in response to drought treatments, and to maintain integrity and stability of leaf cell membranes as a physiological indicator of drought stress tolerance. Leaf sampling and conductance measurements were carried out weekly for three consecutive weeks starting at the end of the sixth week after the drought tolerance treatments commenced. For each of the *Desmodium* species grown under the three water regimes, the uppermost fully expanded leaf trifoliate was selected and the three leaflets individually cut, excluding the petioles. Each set of the harvested leaflets were immediately placed and covered in 50-ml eppendorf plastic tubes to minimize water loss. Fresh weights of the sampled leaflet sets were recorded to enable expression of electrolyte measurements per unit of leaf biomass. Each of the three leaflets in a sample set was then cut into four leaf quarters and then placed back into the eppendorf tubes where they were rinsed three times with distilled deionized water to remove electrolytes adhering on the leaf surfaces. After the last rinse, 20 mL of distilled deionized water was added to 50-ml eppendorf plastic tubes containing the leaf quarters. For each treatment combination, four leaf samples were taken. The tubes were covered with lids and shaken on an orbital shaker at 144 revolutions per minute (rpm) for 2 h after which initial conductance (C_*a*_) was measured using a conductivity meter (Model WTW Cond3110 fitted with a tetracon 325 electrode; WTW 82362 Wellheim, Germany). The units of conductance measurements were microsvedbergs per centimetre (mS cm^−1^). After the initial conductance measurements were taken, the leaf segments were boiled by placing the tubes on a 100 °C water bath for 15 min followed by shaking on an orbital shaker at 144 rpm for another 2 h and then measuring the conductance of the boiled leaf tissues (C_*b*_) after cooling down to room temperature. The first conductance measurement indicated free leaf electrolytes outside the leaf cell membrane (apoplast) while second conductance measurement was the total leaf conductance which indicated total leaf electrolyte status comprised of electrolytes in the apoplast and electrolytes previously bound within the leaf cell membrane (symplast) but thereafter released as a result of aggravated membrane damage arising from exposure of the leaf segments to the hot water bath. To allow for direct comparison of apoplast, symplast and total leaf electrolyte status of the *Desmodium* species evaluated, the leaf segments were oven-dried to constant weight at 85 °C for 24 h and the dry matter weights were used to standardize the conductance measurements to per unit weight of leaf dry matter. Supplementary measurements were conducted to confirm that the soaking and shaking of the leaf segments did not significantly change total leaf dry matter.

### 2.4 Evaluation of striga suppression ability of the *Desmodium* species

The selected *Desmodium* species, together with a drought-tolerant species, *D. intortum*, were evaluated under pot conditions in the screenhouse and field conditions, both on-station and in farmers’ fields, to establish their ability to suppress striga and subsequent effects on grain yields.

#### 2.4.1 Screenhouse pot trials

Screenhouse trials were carried out at *icipe*-TOC using methodologies adapted from Khan et al. (2002). The allelochemical activity of *Desmodium* spp. against striga parasitism on maize was tested by irrigating maize grown in autoclaved striga infested soil with an aqueous solution of chemical components, eluting from established *Desmodium* plants. Pots (20 cm) containing the *Desmodium* species, were placed on shelves and received distilled water at a rate of 1.25 ml/min, thus allowing the flow of water by gravity through the *Desmodium* roots and into pots containing maize situated below. Pots bearing soil alone were used as a negative control. These lower pots were inoculated with approximately 3000 striga seeds/pot and were planted with a striga-susceptible hybrid maize variety, WH505, from Western Seed Company Kenya (Kitale, Kenya). Data on emerged striga were assessed at six weeks after emergence of maize.

#### 2.4.2 On-station field experiment

Field trials were conducted during the long (March–August) and short (October–January) rainy seasons of 2015 using a striga-susceptible, medium maturity, commercial sorghum hybrid, Gadam Hamam, Kenya Seed Company, Kitale, Kenya, recommended for mid-altitude regions (Khan et al., 2002). The sorghum was intercropped in alternate rows with one of the four species of the *Desmodium*, and was planted at an inter-row spacing of 60 cm and intra-row spacing of 30 cm. Alternatively the *Desmodium* was planted through a drilling system in furrows between the rows of sorghum. A control plot of sorghum monocrop was included. The four treatments were randomised and planted in four replications in experimental plots measuring 6 m by 6 m. In all plots, phosphorus in form of di-ammonium phosphate (DAP) was applied at planting at the rate of 60 kg/ha, while nitrogen was applied in the form of calcium ammonium nitrate (CAN), at the rate of 60 kg/ha, 6 weeks later. Plots were kept weed free by hand weeding except for striga throughout the growing season.

Striga infestation was assessed at 8 and 12 weeks after emergence (WAE) of sorghum by counting the number of emerged striga from 50 randomly selected plants per plot. These counts were taken from within a radius of 15 cm around the base of each sorghum plant. The data were expressed as the number of emerged striga per plot. At full physiological maturity, all sorghum plants in each experimental plot were harvested and grain yield converted into tones/ha at 12% moisture content.

#### 2.4.3 On-farm field experiment

On-farm trials were conducted during the long and short rainy seasons of 2015 at two sites in Homabay county, Nuani (0°36.44′S, 34°23.58′E) and Oyiengo (0°34.98′S, 34°22.47′E) to evaluate performance of the selected *Desmodium* species on striga suppression under farmers’ conditions. A similar set up was adopted where plots measuring 6 m by 6 m were established and planted as above, in randomised plots in four replications. Emergence of striga was only assessed at 12 WAE of sorghum and data expressed as the number of emerged striga per plot. Similarly, all sorghum plants in each experimental plot were harvested at full physiological maturity of the crop, and grain yield converted into tones/ha at 12% moisture content.

### 2.5 Data analysis

Data on survival of *Desmodium* species exposed to drought, leaf water content and leaf electrolyte conductance were compared by analysis of variance (ANOVA) using the generalized linear model. Striga count data at peak emergence were utilized for analyses; 6 WAE of maize in the screenhouse pot experiments, 8 WAE of maize in the on-farm experiments, and 8 and 12 WAE of maize in the on-farm experiments. Striga counts were log transformed [log10(x +1)] prior to analysis to normalize the data and stabilize the variance. To avoid possible bias in estimates due to correlated errors in the striga counts repeatedly collected from the same experimental plots, a generalized least squares model was used to examine the effect of treatments and season (time) on log transformed striga counts. ANOVA was performed to examine the effects of treatments and seasons, and their interactions, on maize and sorghum yields. Tukey's honestly significant difference test was used to separate treatment means. All data analyses were conducted in R version 3.1.1 statistical software (R Development Core Team, 2014), with significance level set at α = 0.05 for all analyses.

## 3 Results

### 3.1 Screening *Desmodium* species for drought tolerance

#### 3.1.1 Survival under drought stress

**Fig. 1:**
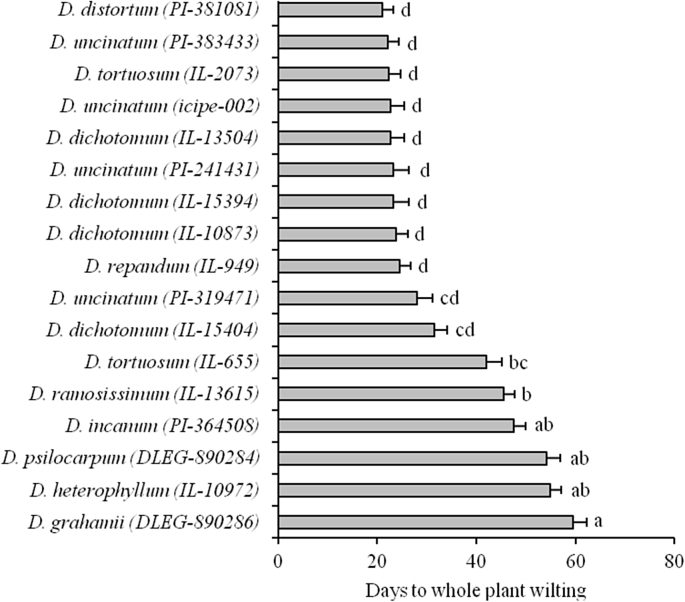
Mean (±SE) Duration to whole plant wilting of 17 accessions of various *Desmodium* species during exposure to severe drought stress conditions in semi-restricted soil columns over a period of ten weeks. Each accession was replicated six times. Bars bearing different letters are significantly different (Tukeys HSD test, α = 0.05).

3.1 One accession of *D. dichotomum* (IL-15404), *D. tortuosum* (IL-655), *D. ramosissimum* (IL-13615), *D. incanum* (PI-364508), *D. psilocarpum* (DLEG-890284), *D. heterophyllum* (IL-10792) and *D. grahamii* (DLEG-890286) withstood significantly longer periods of drought in comparison to the accessions of *D. uncinatum* as control checks (Fig. 1). With the exception of *D. tortuosum,* these species withstood drought at least two times longer than the checks. Indeed, *D. grahamii* survived for about two months (60 days) without water. All the accessions of *D. uncinatum* survived less than one month in absence of watering, with the best performing accession, *D. uncinatum* (PI-319471), only surviving for about 28 days.

#### 3.1.2 Leaf water content

**Fig. 2:**
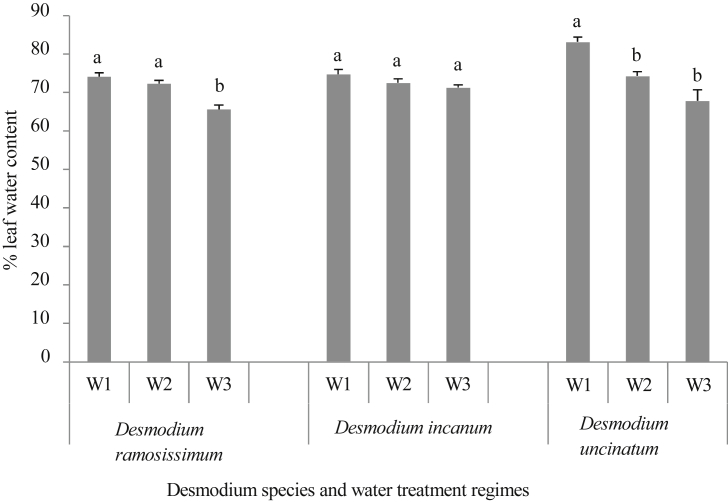
Mean (±SE) % leaf water content of *D. ramosissimum*, *D. incanum* and *D. uncinatum* as affected by six weeks of exposure to moderate (W2) and severe (W3) drought stress conditions and a well-watered control (W1) in restricted soil columns. Means are averages of four replicates. For a given species, bars marked by different letters are significantly different (Tukeys HSD test, α = 0.05).

Leaf water content ranged from 76 to 85% for the four *Desmodium* species for the control treatment in which the plants were adequately watered (Fig. 2). Severe drought stress significantly reduced leaf water content in all the species, except *D. incanum*, when measured in relation to the corresponding control treatments. After six weeks of exposure to severe drought treatment, leaf water content reduced by 41%, 14%, 7%, and 26% for *D. repandum*, *D. ramosissimum*, *D. incanum* and *D. uncinatum*, respectively. Despite the low levels of leaf water loss in *D. incanum* and *D. ramosissimum*, all leaves remained intact under the severe drought treatment. For the moderate drought stress treatment, leaf water content reduced by 9%, 4%, 2%, and 10% for *D. repandum*, *D. ramosissimum*, *D. incanum* and *D. uncinatum*, respectively. The drop in leaf water content was only significant for *D. repandum* and *D. uncinatum*, which shows that these species had reduced water leaf content even when subjected to moderate drought stress conditions.

### 3.2 Leaf electrolyte conductance and cell membrane stability

**Fig. 3:**
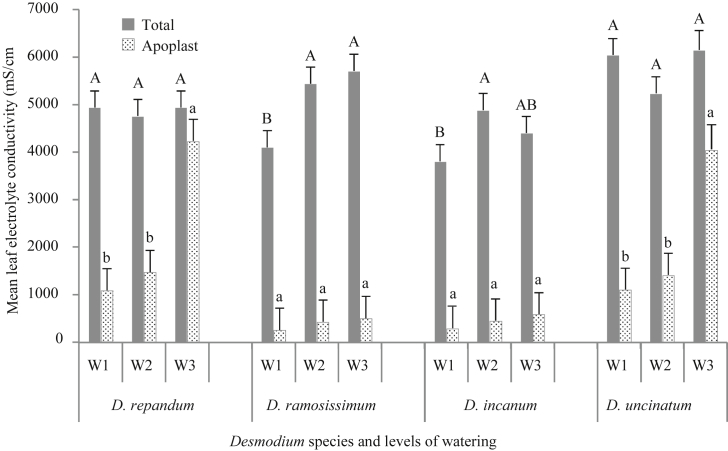
Mean (±SE) leaf electrolyte conductivity of *D. repandum*, *D. ramosissimum*, *D. incanum* and *D. uncinatum* as affected by six weeks of exposure to moderate (W2) and severe (W3) drought stress conditions as well as a well-watered control (W1). The dotted bars indicate conductivity attributed to free electrolytes outside leaf cell membrane (apoplast) while the gray bars indicate conductivity attributed to total leaf electrolytes (electrolytes in the apoplast and symplast). Means represent averaged of four replicates. For each category of electrolytes in a given species, bars marked by different letters are significantly different (Tukeys HSD test, α = 0.05).

Results indicated a clear increase in total leaf electrolyte conductance during exposure to the drought stress treatments on *D. ramosissimum* and *D. incanum* (Fig. 3), with exposure to moderate and severe drought stress treatments significantly increasing total leaf conductance in comparison to the control. Under the severe drought treatment, total leaf electrolyte conductivity increased by 39% in *D. ramosissimum* and 16% in *D. incanum*, while in *D. uncinatum* the increase in electrolyte concentration was only 2%. Leaf apoplast electrolyte fractions in *D. incanum* and *D. ramosissimum* did not significantly increase despite increased accumulation of total leaf electrolytes during exposure to the severe drought stress treatment, in contrast to drastic increases in *D. repandum* and *D. uncinatum,* 289% and 267% respectively.

### 3.3 Evaluation of striga suppression ability of the *Desmodium* species

#### 3.3.1 Screenhouse pot trials

**Fig. 4:**
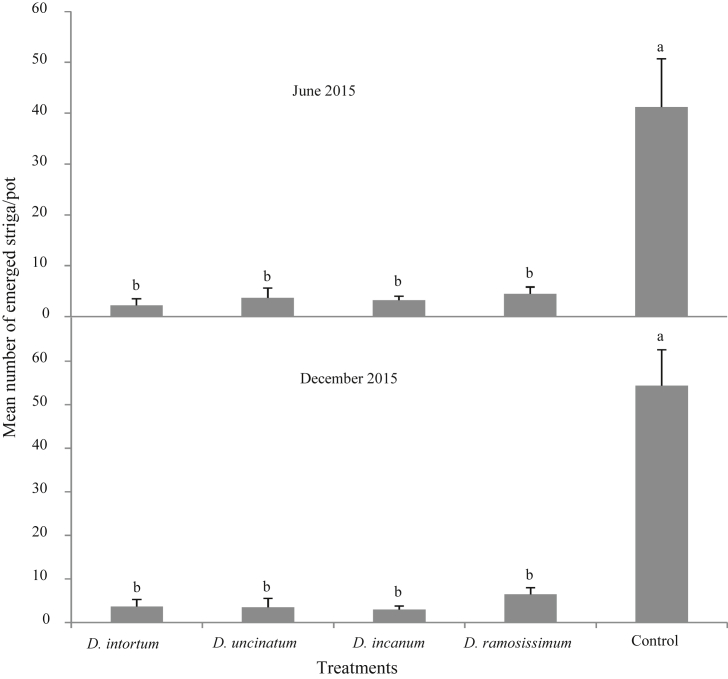
Mean (±SE) number of emerged striga plants per pot treated with and without *Desmodium* root exudates (n = 18). Within a graph, bars marked by different letters are significantly different (Tukeys HSD test, α = 0.05).

Emergence of striga was significantly suppressed in the pots irrigated with the root exudates of all the *Desmodium* species tested compared with irrigation through soil alone during both study periods, June and December 2015 (June: F_4,15_ = 11.08; P < 0.001; December: F_4,15_ = 9.76; P < 0.001, Fig. 4). There was over 90% suppression of striga emergence on root exudate treated maize relative to the distilled water control, showing the root exudates of the drought-tolerant *Desmodium* species were effective in suppressing striga parasitism.

#### 3.3.2 On-station field studies

The *Desmodium* species equally and effectively suppressed striga infestations in both seasons (Table 1). Mean emerged striga counts were significantly lower in all sorghum-*Desmodium* intercrops during the long (8WAE: F_4,15_ = 9.48; P < 0.001; 12WAE: F_4,15_ = 17.05; P < 0.001) and short (8WAE: F_4,15_ = 9.70; P < 0.001; 12WAE: F_4,15_ = 19.32; P < 0.001) rainy seasons relative to the sorghum monocrop. However, there were no significant differences between the intercrops, indicating effectiveness of the drought-tolerant *Desmodium* spp. in suppressing striga. Emerged striga counts ranged between 7 and 33 plants per plot with sorghum-*D. uncinatum* and sorghum-*D. incanum* intercrops, respectively, during the short rainy season. However, they ranged between 235 and 315 plants per plot with the sorghum monocrop during the long rainy season. These observations were associated with significantly higher grain yields in the *Desmodium* intercrops than in sorghum monocrop during both cropping seasons (Long rainy season; F_4,15_ = 17.42; P < 0.001; Short rainy season; F_4,15_ = 9.40; P < 0.001). Grain yields of sorghum ranged between 2.9 t/ha and 3.96 t/ha with *D. ramosissimum* and *D. intortum* intercrops during the short and long rainy seasons, respectively. However, with sorghum monocrop the grain yields only ranged between 1.2 and 1.3 t/ha during the short and long rainy seasons, respectively. There were no significant differences between the *Desmodium* spp. intercrops with regards to grain yields, indicating the drought-tolerant species performed as effectively as the conventional species in improving grain yields.

**Table 1:** Mean (±SE) number of striga counts in sorghum plots planted in sole stands or intercropped with various *Desmodium* spp. at *icipe*-Thomas Odhiambo Campus, western Kenya during the long and short rainy seasons of 2015.

Treatment	Mean striga counts LR 2015		Grain yields (t/ha)	Mean striga counts SR 2015		Grain yields (t/ha)
	8th WAE	12th WAE		8th WAE	12th WAE	
Sorghum-*D. intortum*	15.5(6.3)b	27.2(5.9)b	3.96(0.21)a	10.5(4.8)b	30.7(11.7)b	3.34(0.17)a
Sorghum-*D. uncinatum*	26.0(9.0)b	24.3(10.6)b	3.33(0.20)a	7.0(3.2)b	15.5(6.3)b	3.32(0.29)a
Sorghum-*D. incanum*	26.7(8.5)b	31.7(6.4)b	3.26(0.40)a	14.8(3.4)b	32.7(12.0)b	3.0(0.34)a
Sorghum-*D. ramosissimum*	28.0(11.5)b	31.5(9.0)b	3.40(0.18)a	24.2(9.3)b	30.5(9.3)b	2.93(0.38)a
Sorghum monocrop	235.5(27.0)a	315.2(21.1)a	1.32(0.13)b	305.8(27.6)a	237.8(62.0)a	1.23(0.18)b

Within a sample period and cropping season (each column), means marked by different letters are significantly different (p < 0.05).

#### 3.3.3 On-farm field studies

The *Desmodium* spp. effectively suppressed striga under farmers' field conditions at Oyiengo and Nuani (Table 2), although striga counts were generally lower in farmers’ fields that at *icipe*-ITOC during both cropping seasons. Mean emerged striga counts were significantly lower in the various *Desmodium* intercrops compared to the sorghum monocrop plots at both sites during both seasons (Oyiengo, long rainy season: F_4,15_ = 19.94; P < 0.001; short rainy season: F_4,15_ = 5.4; P = 0.007; Nuani, long rainy season: F_4,15_ = 9.35; P < 0.001; short rainy season: F_4,15_ = 23.04; P < 0.001); except at Oyiengo during the short rainy season when the differences in striga counts were not significant between the sorghum monocrop and the two drought-tolerant *Desmodium* spp. intercrops, although the striga counts were relatively higher in the former. At Oyiengo, these counts ranged between 10 and 61 plants per plot with *D. intortum* and *D. ramosissimum* intercrops during the long and short rainy seasons, respectively. These counts were much lower in Nuani, where they ranged between 1 and 13 plants per plot with *D. intortum* and *D. ramosissimum* intercrops, respectively, during the short rainy season. However, they ranged between 49 at Nuani during the long rainy season and 173 plants per plot at Oyiengo during the short rainy season, with the sorghum monocrop.

**Table 2:** Mean (±SE) number of striga counts in sorghum plots planted in sole stands or intercropped with various *Desmodium* spp. in smallholder farmers' fields at two sites, Oyiengo and Nuani, in Homabay County, western Kenya during the long and short rainy seasons of 2015.

Treatment	Mean number of emerged striga				Sorghum grain yields (t/ha)			
	Oyiengo Site		Nuani Site		Oyiengo Site		Nuani Site	
	LR 2015	SR 2015	LR 2015	SR 2015	LR 2015	SR 2015	LR 2015	SR 2015
Sorghum-*D. intortum*	10.2(1.4)c	39.5(17.9)b	2.5(1.3)b	0.8(0.5)c	4.1(0.1)a	3.3(0.2)a	3.1(0.1)a	2.6(0.2)a
Sorghum-*D. uncinatum*	19.5(4.8)bc	24.0(10.4)b	2.8(1.1)b	11.8(2.8)b	3.9(0.1)ab	3.2(0.2)a	2.9(0.1)ab	2.8(0.3)a
Sorghum-*D. incanum*	22.7(6.0)bc	60.0(11.6)ab	6.0(1.9)b	5.0(3.0)bc	3.7(0.1)b	2.9(0.2)a	2.6(0.1)b	2.5(0.3)a
Sorghum-*D. ramosissimum*	36.7(9.1)b	61.0(15.0)ab	3.8(2.8)b	13.0(1.2)b	3.7(0.1)b	3.1(0.2)a	2.5(0.1)b	2.6(0.2)a
Sorghum monocrop	128(8.0)a	173(45.5)a	49(11.7)a	85.2(8.7)a	1.9(0.1)c	1.9(0.1)b	1.8(0.1)c	1.7(0.1)b

Within a site and cropping season (each column), means marked by different letters are significantly different (p < 0.05).

Sorghum grain yields were consistently and significantly higher with the *Desmodium* intercrops relative to the sorghum monocrop at both sites during both cropping seasons (Oyiengo, long rainy season: F_4,15_ = 118.31; P < 0.001; short rainy season: F_4,15_ = 8.32; P = 0.001; Nuani, long rainy season: F_4,15_ = 22.67; P < 0.001; short rainy season: F_4,15_ = 3.35; P < 0.001) (Table 2). They ranged between 2.5 t/ha and 4.1 t/ha with the *Desmodium* intercrops, and 1.7 t/ha and 1.9 t/ha with the sorghum monocrop.

## 4 Discussion and conclusions

Drought stress, often linked with high atmospheric temperatures, is a major challenge to crop production in SSA where agriculture is mainly rain-fed. Indeed warming in the region is expected to be greater than the global average, with drought gradually becoming a widespread phenomenon (Heisey and Edmeades, 1999, IPCC, 2007). Rainfall is also becoming less predictable (Downing, 1992), associated with increased growing season temperatures and frequency of crop failures. Such environmental changes are projected to aggravate the distribution and increase the competitiveness of striga (Rodenburg and Meinke, 2010). There is thus a need to adapt striga management technologies, such as the *Desmodium*-based approaches, to dry conditions.

Following exposure of the *Desmodium* accessions to 10 continuous weeks of drought treatment, two categories of drought tolerance responses emerged among the species that withstood longer periods of drought relative to the check. In the first category, which included accessions such as *D. tortuosum* (IL-655), exposure of the plants to drought stress led to accelerated leaf senescence and abscission of majority of leaves. This reaction was possibly a response of the plants to reduce canopy size and lower evapo-transpiration water losses. Although this trait enables the plants to withstand dry conditions (Rivero et al., 2007), such reactions reduce the plant's overall photosynthetic competence and capacity to synthesize and emit essential leaf volatiles needed for different physiological and ecological functions of importance to the plant, such as cereal stemborer control (Khan et al., 2000). The drought-tolerant species with accelerated leaf senescence and abscission were considered less desirable and therefore not targeted for selection in the screening. In the second category, represented by *D. ramosissimum* and *D. incanum,* the accessions retained and maintained most of their leaves despite exposure to severe drought stress conditions. This benefits the plant through continued photosynthesis of carbon assimilates needed to meet the energy requirements of biological nitrogen fixation processes, production of root exudates that inhibit striga, production and emission of leaf volatiles that repel cereal stemborers, and accumulation of above ground biomass that could be utilized as cover for soil and as fodder for livestock.

In subsequent screening, *D. ramosissimum* and *D. incanum* recorded significantly lower leaf water loss but showed significant increases in total leaf electrolyte conductance during exposure to drought stress. This possibly arises as part of an osmotic adjustment process that in general involves plant accumulation of compatible solutes including sugars, ions and amino acids in response to drought conditions. The increased accumulation of solutes could lower leaf osmotic potential and allow movement of water into the leaf cells, thereby maintaining turgor potential and increasing tissue tolerance to low soil water conditions. The accumulated solutes also sequester water molecules, protect cell membranes and protein complexes and allow continued cell metabolic functioning (Chaves et al., 2003). Moreover, leaf apoplast electrolyte fractions in *D. incanum* and *D. ramosissimum* did not significantly increase despite increased accumulation of total leaf electrolytes during exposure to the severe drought stress treatment, suggesting that these species maintained their leaf cell membrane stability which not only enhanced retention but also enabled accumulation of electrolytes within the symplast during exposure to severe drought.

Following selection of the drought-tolerant species above, the next step was to evaluate their performance in suppression of striga. Results indicated significant reductions in the number of emerged striga in the pots watered through desmodium roots in the screenhouse experiment, showing the root exudates of the tested *Desmodium* species were effective in inhibiting striga parasitism. Similarly, striga infestation was significantly lower in plots intercropped with the *Desmodium* species, indicating effective control of this noxious weed under field conditions. Notably, the drought-tolerant *Desmodium* spp. gave similar levels of weed control as those obtained with both *D. uncinatum* and *D. intortum*, species that have shown effective control of the weed and have been widely adopted by smallholder farmers in eastern Africa (Khan et al., 2014, Midega et al., 2014, Midega et al., 2015). These results demonstrate the incremental capability of *Desmodium* species to suppress striga, resulting in significant increases in crop yields, and thus contribute to the accumulating body of knowledge on the importance of intercropping with *Desmodium* in smallholder cereal farming systems in the region.

Striga inhibition by *Desmodium* spp. is achieved through an allelopathic mechanism and benefits derived from increased availability of nitrogen and soil shading (e.g. Khan et al., 2002, Tsanuo et al., 2003). The allelopathic effect involves root exudates, produced independently of the presence of striga, which are responsible for the dramatic inhibition of striga in intercrop with cereal crops. *Desmodium* root exudates contain novel isoflavanones, some of which stimulate suicidal germination of striga seeds while others, and a group of *C*-glycosylflavones, inhibit radicle growth (Tsanuo et al., 2003, Hooper et al., 2009, Hooper et al., 2010). This combination thus provides a novel means of *in situ* reduction of striga seed bank in the soil even with the presence of graminaceous host plants in the proximity. A more recent study by Hooper et al. (2015) revealed that this chemistry exists in a number of *Desmodium* spp., demonstrating that taxonomically related *Desmodium* species produce similar root exudate chemistry to the *Desmodium* species known to be effective in the field (*D. uncinatum* and *D. intortum*), and so are potential intercrops for inhibition of striga parasitism of subsistence cereals in dry agronomic environments.

There is evidence that effects of striga are most severe in degraded and infertile soils (Reda et al., 2005), therefore rehabilitation of soil health has an important role in an integrated striga management strategy for smallholder farming systems in SSA. In addition to the direct control of striga, perennial legumes have the potential to fix substantial amounts of nitrogen, and *Desmodium* spp. are no exception. Indeed, *D. intortum* has been reported to fix over 300 kg N/ha per year under optimum conditions (Whitney, 1966). The improvements in crop productivity observed under *Desmodium* intercrops results from a range of factors beyond nitrogen fixation and soil organic matter increases; *Desmodium* conserves soil moisture (Khan et al., 2002) and improves arthropod diversity and activity (Midega et al., 2008). These results thus demonstrate that *Desmodium* spp. increase options available to farmers that fall in different socio-economic strata, including subsistence farmers, in different agro-ecological zones in SSA, many of whom practice mixed cropping and keep livestock (*Desmodium* being valuable protein source for livestock) in degraded environments, and where intercropping is already an integral part of farming systems (Abate et al., 2000).

In conclusion, *D. incanum* and *D. ramosissimum* are selected as the most promising species among the *Desmodium* species accessions evaluated for tolerance to drought stress because of leaf retention and maintenance of leaf function for longer periods during exposure to drought stress conditions. The two species showed capacity to increase total leaf electrolytes as part of an osmotic adjustment process while restricting the accumulated electrolytes within the symplast and keeping constant the electrolyte fractions within the leaf cell apoplast, indicating a sustained competence of leaf cell membranes. These species also had desirable plant phenotypes and accumulated more above ground biomass. Notably, they suppressed striga infestation, both under controlled and farm conditions, suggesting that the beneficial allelochemical traits released by the roots of *Desmodium* is common among such congeneric species. However, there is need to evaluate their root exudate activity against striga in arid conditions that may present different biotic and environmental challenges, and where moisture in the rhizosphere may affect allelochemical production and release. Additionally, there is need to evaluate potential role of these *Desmodium* spp. in improving soil health and possible beneficial effects in mitigation of climate change-driven effects such as soil degradation that are prevalent in SSA.

## References

[bib1] Abate T., van Huis A., Ampofo J.K.O. (2000). Pest management strategies in traditional agriculture: an African perspective. Ann. Rev. Entomol..

[bib2] Abraham E.M., Huang B., Bonos S.A., Meyer W.A. (2004). Evaluation of drought resistance for Texas bluegrass, Kentucky bluegrass, and their hybrids. Crop Sci..

[bib3] Bebawi F.F., Metwali E.M. (1991). Witch-weed management by sorghum–Sudan grass seed size and stage of harvest. Agron. J..

[bib4] Bouchabke O., Chang F., Simon M., Voisin R., Pelletier G. (2008). Natural variation in Arabidopsis thaliana as a tool for highlighting differential drought responses. PLoS One.

[bib5] Chaves M.M., Maroco J.P., Pereira J.S. (2003). Understanding plant responses to drought: from genes to the whole plant. Funct. Plant Biol..

[bib6] Downing T.E. (1992). Climate Change and Vulnerable Places: Global Food Security and Country Studies in Zimbabwe, Kenya, Senegal and Chile.

[bib7] Gethi J.G., Smith M.E., Mitchell S.E., Kresovich S. (2005). Genetic diversity of *Striga hermonthica* and *Striga asiatica* populations in Kenya. Weed Res..

[bib8] Gressel J., Hanafi A., Head G., Marasas W., Obilana A.B., Ochanda J., Souissi T., Tzotzos G. (2004). Major heretofore intractable biotic constraints to African food security that may be amenable to novel biotechnological solutions. Crop Prot..

[bib9] Gurney A.L., Slate J., Press M.C., Scholes J.D. (2006). A novel form of resistance in rice to the angiosperm parasite *Striga hermonthica*. New Phytol..

[bib10] Heisey P.W., Edmeades G.O. (1999). Maize production in Drought-stressed Environments: Technical Options and Research Resource Allocation. http://repository.cimmyt.org/xmlui/bitstream/handle/10883/759/67377.pdf.

[bib11] Hirashima M., Tanaka R., Tanaka A. (2009). Light-independent cell death induced by accumulation of pheophorbide a in *Arabidopsis thaliana*. Plant Cell Physiol..

[bib12] Hooper A.M., Caulfield J.C., Hao B., Pickett J.A., Midega C.A.O., Khan Z.R. (2015). Isolation and identification of Desmodium root exudates from drought tolerant species used as intercrops against *Striga hermonthica*. Phytochemistry.

[bib13] Hooper A.M., Hassanali A., Chamberlain K., Khan Z.R., Pickett J.A. (2009). New genetic opportunities from legume intercrops for controlling *Striga spp*. parasitic weeds. Pest Manage. Sci..

[bib14] Hooper A.M., Tsanuo M.K., Chamberlain K., Tittcomb K., Scholes J., Hassanali A., Khan Z.R., Pickett J.A. (2010). Isoschaftoside, a *C*-glycosylflavonoid from *Desmodium uncinatum* root exudate, is an allelochemical against the development of striga. Phytochemistry.

[bib15] IPCC (2007). Fourth Assessment Report: Synthesis. http://www.ipcc.ch/pdf/assessment-report/ar4/syr/ar4_syr.pdf.

[bib16] Kanampiu F., Friesen D., Gressel J. (2002). CIMMYT unveils herbicide-coated maize seed technology for *Striga* control. Haustorium.

[bib17] Kanampiu F.K., Kabambe V., Massawe C., Jasi L., Friesen D., Ransom J.K., Gressel J. (2003). Multi-site, multi-season field tests demonstrate that herbicide seed-coating herbicide-resistance maize controls *Striga* spp. and increases yields in several African countries. Crop Prot..

[bib18] Khan Z.R., Hassanali A., Overholt W., Khamis T.M., Hooper A.M., Pickett A.J., Wadhams L.J., Woodcock C.M. (2002). Control of witchweed *Striga hermonthica* by intercropping with *Desmodium* spp., and the mechanism defined as allelopathic. J. Chem. Ecol..

[bib19] Khan Z.R., Midega C.A.O., Amudavi D.M., Hassanali A., Pickett J.A. (2008). On-farm evaluation of the ‘push–pull’ technology for the control of stemborers and striga weed on maize in western Kenya. Field Crops Res..

[bib20] Khan Z.R., Midega C.A.O., Pittchar J.O., Murage A.W., Birkett M.A., Bruce T.J.A., Pickett J.A. (2014). Achieving food security for one million sub-Saharan African poor through push–pull innovation by 2020. Phil. Trans. R. Soc. B.

[bib21] Khan Z.R., Pickett J.A., Van den Berg J., Wadhams L.J., Woodcock C.M. (2000). Exploiting chemical ecology and species diversity: stemborer and *Striga* control for maize and sorghum in Africa. Pest Manage. Sci..

[bib22] Midega C.A.O., Bruce T.J.A., Pickett J.A., Pittchar J.O., Murage A., Khan Z.R. (2015). Climate-adapted companion cropping increases agricultural productivity in East Africa. Field Crops Res..

[bib23] Midega C.A.O., Khan Z.R., Van den Berg J., Ogol C.K.P.O., Dippenaar-Schoeman A.S., Pickett J.A., Wadhams L.J. (2008). Response of ground-dwelling arthropods to a ‘push–pull’ management system: spiders as an indicator group. J. Appl. Entomol..

[bib24] Midega C.A.O., Salifu D., Bruce T.J., Pittchar J., Pickett J.A., Khan Z.R. (2014). Cumulative effects and economic benefits of intercropping maize with food legumes on *Striga hermonthica* infestation. Field Crops Res..

[bib25] Oldeman L.R., Hakkelling R.T.A., Sombroek W.G. (1990). World map of the status of human- induced soil degradation (GLASOD) with explanatory note. United Nations Environmental Program, Nairobi, Kenya.

[bib26] R Core Team (2014). R: a Language and Environment for Statistical Computing. http://www.R-project.org/.

[bib27] Reda F., Verkleij J.A.C., Ernst W.H.O. (2005). Intercropping for the improvement of sorghum yield, soil fertility and striga control in the subsistence agriculture region of Tigray (Northern Ethiopia). J. Agron. Crop Sci..

[bib28] Rivero R.M., Kojima M., Gepstein A., Sakakibara H., Mittler R., Gepstein S., Blumwald E. (2007). Delayed leaf senescence induces extreme drought tolerance in a flowering plant. Proc. Natl. Acad. Sci..

[bib29] Rodenburg, J., Meinke, H., 2010. Adapting weed management in rice to changing climates. Second Africa Rice Congress, Bamako, Mali, 22–26 March 2010: Innovation and Partnerships to Realize Africa’s Rice Potential. http://www.africaricecenter.org/workshop/ARC/5.6%20Rodenburg%20fin.pdf.

[bib30] Sivakumar M.V.K. (1993). Global climate change and crop production in the Sundano-Sahelian zone of west Africa.

[bib31] Tenebe V.A., Kamara H.M. (2002). Effect of *Striga hermonthica* on the growth characteristics of sorghum intercropped with groundnut varieties. J. Agron. Crop Sci..

[bib32] Tsanuo M.K., Hassanali A., Hooper A.M., Khan Z.R., Kaberia F., Pickett J.A., Wadhams L. (2003). Isoflavanones from the allelopathic aqueous root exudates of *Desmodium uncinatum*. Phytochemistry.

[bib33] Whitney A.S. (1966). Nitogen fixation by three tropical forage legumes and the utilization of legume-fixed nitrogen by their associated grasses. Herb. Abstr..

